# Differential effect of maternal diet supplementation with α-Linolenic adcid or n-3 long-chain polyunsaturated fatty acids on glial cell phosphatidylethanolamine and phosphatidylserine fatty acid profile in neonate rat brains

**DOI:** 10.1186/1743-7075-7-2

**Published:** 2010-01-14

**Authors:** Frédéric Destaillats, Corinne Joffre, Niyazi Acar, Florent Joffre, Jean-Baptiste Bezelgues, Bruno Pasquis, Cristina Cruz-Hernandez, Serge Rezzi, Ivan Montoliu, Fabiola Dionisi, Lionel Bretillon

**Affiliations:** 1 Nestlé Research Center, Vers-chez-les-Blanc, Lausanne, Switzerland; 2 Eye and Nutrition Research Group, INRA, UMR1129 FLAVIC, F-21000 Dijon, France; 3 Omega 21, 10 route de Flacey 21380 Marsannay le Bois, France

## Abstract

**Background:**

Dietary long-chain polyunsaturated fatty acids (LC-PUFA) are of crucial importance for the development of neural tissues. The aim of this study was to evaluate the impact of a dietary supplementation in n-3 fatty acids in female rats during gestation and lactation on fatty acid pattern in brain glial cells phosphatidylethanolamine (PE) and phosphatidylserine (PS) in the neonates.

**Methods:**

Sprague-Dawley rats were fed during the whole gestation and lactation period with a diet containing either docosahexaenoic acid (DHA, 0.55%) and eicosapentaenoic acid (EPA, 0.75% of total fatty acids) or α-linolenic acid (ALA, 2.90%). At two weeks of age, gastric content and brain glial cell PE and PS of rat neonates were analyzed for their fatty acid and dimethylacetal (DMA) profile. Data were analyzed by bivariate and multivariate statistics.

**Results:**

In the neonates from the group fed with n-3 LC-PUFA, the DHA level in gastric content (+65%, P < 0.0001) and brain glial cell PE (+18%, P = 0.0001) and PS (+15%, P = 0.0009) were significantly increased compared to the ALA group. The filtered correlation analysis (P < 0.05) underlined that levels of dihomo-γ-linolenic acid (DGLA), DHA and n-3 docosapentaenoic acid (DPA) were negatively correlated with arachidonic acid (ARA) and n-6 DPA in PE of brain glial cells. No significant correlation between n-3 and n-6 LC-PUFA were found in the PS dataset. DMA level in PE was negatively correlated with n-6 DPA. DMA were found to occur in brain glial cell PS fraction; in this class DMA level was correlated negatively with DHA and positively with ARA.

**Conclusion:**

The present study confirms that early supplementation of maternal diet with n-3 fatty acids supplied as LC-PUFA is more efficient in increasing n-3 in brain glial cell PE and PS in the neonate than ALA. Negative correlation between n-6 DPA, a conventional marker of DHA deficiency, and DMA in PE suggests n-6 DPA that potentially be considered as a marker of tissue ethanolamine plasmalogen status. The combination of multivariate and bivariate statistics allowed to underline that the accretion pattern of n-3 LC-PUFA in PE and PS differ.

## Background

Nutrient supply is of crucial importance in the maturation and functional development of the central nervous system [1]. Long-chain polyunsaturated fatty acids (LC-PUFA) such as arachidonic acid (ARA, C20:4n-6) and docosahexaenoic acid (DHA, C22:6n-3) are major constituents of membrane phospholipids in the brain and the retina [2]. It has been shown that the level of DHA in retinal phospholipids has a strong impact on visual transduction processes and subsequently to visual function assessed by electroretinography [3,4]. Beside their structural implications, biologically active derivatives of LC-PUFA such as neuroprotectins and resolvins, are involved in neuroprotection, signalling and maintaining or lowering the inflammatory response [5-7].

ARA and DHA can be synthesized from dietary essential fatty acids: linoleic (18:2n-6, LA) and α-linolenic (ALA, C18:3n-3) acids, respectively, through a series of elongation and desaturation steps catalyzed by the same key enzymes, namely Δ6- and Δ5-desaturases [2]. Therefore, the level of LA and ALA in the diet is very important to ensure adequate deposition of LC-PUFA in membrane phospholipids in the neonate [2,8-10]. However, it has been demonstrated in many neonate animal models that the rate of conversion of ALA to DHA is not appropriate to sustain optimal DHA accretion in the brain and the retina just after birth [11-13]. Nevertheless, the DHA supply is a key factor for the ocular and cerebral development steps that take place during the last trimester of pregnancy in humans [14]. It has been demonstrated that the maternal conversion rate of ALA to DHA is not sufficient to fulfil the needs of the foetus [14]. Therefore, it is currently recommended to consume LC-PUFA rich foods during pregnancy and lactation in order to deliver the optimal level of DHA to the foetus [14]. It has been demonstrated that maternal supplementation with n-3 LC-PUFAs might positively influence cognitive performance in infants [15] but the number of conclusive reports in this area is limited.

In a recent study, Bowen and Clandinin [10] clearly demonstrated that dietary supply of DHA is more efficient than ALA to increase the DHA level in brain (cerebrum plus cerebellum) glial cell phospholipids in rat neonates during lactation [10]. Glial cells are important support to neuronal cells and especially for neurotransmission, neuroprotection, energy maintenance and supply of key elements [1]. It has been shown that glial cells are able to convert fatty acids and synthesize LC-PUFAs while it is not the case for neuronal cells [16]. The present study has been carried out with the same experimental design except that the nutritional intervention started earlier, *i.e*. during gestation, and was maintained throughout lactation. This design allows to study the effect of the maternal nutrition along all the stages of neurodevelopment that started *in utero *and are almost completed at weaning in rat [1]. Maternal milk compositions have been assessed by analyzing gastric content of neonates two weeks after birth. Analysing the fatty acid composition of the gastric content gives insight on the maternal milk composition. The fatty acid composition of phosphatidylethanolamine (PE) and phosphatidylserine (PS) brain glial cells has been assessed in 14 days rat neonates. A combination of multivariate and bivariate statistics has been used to better exemplify how n-3 fatty acids, supplied in the maternal diet as precursor (ALA) or LC-PUFA (EPA and DHA), impact fatty acid pattern in brain glial cell PE and PS, the two main classes of phospholipids rich in LC-PUFA.

## Materials and methods

### Animals and experimental design

The protocol was conducted following the *Guidelines for the Care and Use of Experimental Animals *and approved by the local ethical committee. Sprague Dawley rats (11 weeks of age) were mated for a period of 10 days (1 male + 1 female per cage) at INRA (Animal Breeding House, INRA Research Center, Dijon, France) under controlled conditions for light (lights on, 7:00 AM-7:00 PM), temperature (22 ± 1°C) and hygrometry (55-60%). Water and food were supplied *ad libitum *to the animals from the first day of the mating period until the end of the suckling period. At day 1 after parturition, the number of pups was adjusted to 10 neonates per litter. Mortality of the pups was recorded daily after parturition until the end of the follow-up. Pups were weighed once a week.

### Dietary fatty acid supplementation

The composition of the diets and the n-3 fatty acid content of dietary fat is provided in Table 1. The relative distribution of fatty acid classes was similar to that described by Bowen and Clandinin [10]. Basically, the ALA lipid blend contained rapeseed oil (27.1%), cocoa butter (23.4%), coconut oil (18.7%), high-oleic sunflower oil (15.9%), and sunflower oil (14.8%). The n-3 LC-PUFA lipid blend was composed of rapeseed oil (21.6%), cocoa butter (21.6%), coconut oil (19.2%), high-oleic sunflower oil (17.4%), sunflower oil (15.0%) and fish oil (5.2%).

**Table 1:** Composition of the experimental diets (in g per kg of diet)

Nutrient (in g/kg diet)	ALA	n-3 LC-PUFA	Energy %
			

Lipid blend	200.00	200.00	39.9
Casein	270.00	270.00	23.9
Starch	200.00	200.00	17.7
Sucrose	207.65	207.65	18.4
Non nutritive fiber	50.00	50.00	0.0
Vitamins (mix)	10.00	10.00	0.0
Minerals (mix)	50.85	50.85	0.0
L-methionin	2.50	2.50	0.0
Choline	2.75	2.75	0.0
Inositol	6.25	6.25	0.0

### Tissue collection

Male pups were euthanized at day 14 after birth (n = 24 in each group). Brains (cerebrum plus cerebellum) were excised and placed in ice-cold 0.32 M sucrose as previously described [10,17]. Six brains from pups at day 14 were pooled together for glial cell separation. A total of 4 pools were prepared and used as a starting material for glial cell isolation. The gastric content of pups sacrificed at day 14 after birth was removed and stored at -80°C until further analysis.

### Isolation of brain glial cells from brain samples

Glial cells were purified from the whole brain (cerebrum plus cerebellum) by centrifugation as described in the literature [17]. Briefly, pooled brains were homogenized in 7.5% (w/v) polyvinylpyrrolidone and 10 mM CaCl_2 _at pH 4.7 and 25°C. The homogenate was layered on a two-step sucrose gradient of 1.0 and 1.75 M. After centrifugation at 41,000 g for 30 min at 4°C, glial cells were isolated from the interface of the 1.0 and 1.75 M sucrose layers.

### Lipid Extraction

Lipids were extracted from brain glial cells and gastric content according to the Folch procedure [18].

### Phospholipid separation by high-performance liquid chromatography (HPLC)

Phospholipids from brain glial cell lipid extracts were separated by preparative HPLC using a Lichrosorb Si60 (5 μm, Merck) fitted with a light-scattering detector as described in the literature [19]. Fractions containing PS and PE were collected and stored at -80°C under inert nitrogen-conditions before fatty acid analysis.

### Fatty acid analysis by gas-liquid chromatography (GLC)

Fatty acid methyl esters (FAME) were prepared from gastric contents following the methods of Glass [20] while for the experimental diets and glial cells, the method described by Morrison and Smith [21] was used. FAME samples were analyzed by gas-liquid chromatography using a fused silica capillary column (CP-Sil 88, 100 m × 0.25 mm id, 0.25 μm film thickness, Varian, Les Ulis, France) operating under conditions described elsewhere [22,23].

### Statistical analyses

#### Bivariate statistical analysis

Fatty acid profile of pup's gastric content, PE and PS brain glial cells were analyzed by ANOVA procedure, followed by post-hoc Dunnett's test, using the SAS software (SAS Institute, Cary, USA). Differences were considered as significant at P < 0.05.

#### Principal Components Analysis (PCA)

PCA was used to analyze the fatty acid profile of PE and PS purified from brain glial cell samples. Data pretreatment, correlation analysis and Principal Component Analysis were done on Matlab™ 7.5 (The Mathworks, Inc., MA, USA). In-house written routines were used for data import and visualization, whilst preprocessing, correlation analysis and PCA modeling were done using the PLS-Toolbox v 5.22 (Eigenvector Research Inc., WA, USA).

## Results

### Animal data

Supplementation with n-3 LC-PUFA during gestation did not modify any characteristics of the newborn rats compared to the control group in terms of number of pups per litter, weight and growth of the rats during the first two weeks of age of the pups (Table 2). No significant differences were observed between both groups of animals, neither at the time of parturition nor at weaning.

**Table 2:** Data on reproduction efficiency and weight of animals at birth and during the first two weeks of age.

Parameters	Experimental group	
	
	ALA	n-3 LC-PUFA
		
Mating efficiency (pregnant females/total mated females)	16/18	16/18
Parturition time after starting to mate (days)	24 ± 1	22 ± 1
Total number of newborn male rats (from 16 litters)	105	110
Total number of newborn female rats (from 16 litters)	100	118
Number of newborn rats per litter	12.8 ± 4.5	14.3 ± 3.0
Mean weight of the newborn rat (g)	7.7 ± 1.7	7.1 ± 0.7
Mean weight of the rat at birth after adjusting to 10 rats per litter (g)	7.7 ± 1.5	7.2 ± 0.6
Mortality during the first days of life (n)	1 at 7 days	3 at 7 days
Weight of the rat at day 14 (g)	46.3 ± 3.3	46.0 ±4.3
Brain weight at day 14 (g)	1.37 ± 0.13	1.34 ± 0.09

### Fatty acid composition of the experimental diets

In the present study, lipid represented about 40% of the energy provided by the experimental diets (Table 1). This level reflects the energy coming from lipids in typical US diet and was previously used in similar studies by Bowen and Clandinin [10]. The fatty acid distribution of the experimental diets is provided in Table 3. The ALA diet does not contain any n-3 LC-PUFA while the lipid fraction of the n-3 LC-PUFA experimental diet contains EPA and DHA (0.75 and 0.55% of total fatty acids, respectively). The level of saturated, monounsaturated and polyunsaturated fatty acids were balanced in both experimental diets to *c.a*. 37-38, 42-43 and 20% of total fatty acids, respectively. The n-6 to n-3 fatty acid ratios were 8 and 5 for the ALA and n-3 LC-PUFA experimental diets, respectively (Table 3).

**Table 3:** Fatty acid composition of the experimental diets.

	ALA	n-3 LC-PUFA
C8:0	1.50	1.55
C10:0	1.27	1.33
C12:0	8.70	9.14
C14:0	3.26	3.72
C16:0	10.63	10.96
C17:0	0.08	0.11
C18:0	10.64	10.13
C20:0	0.54	0.51
C22:0	0.37	0.37
C24:0	0.13	0.13
C16:1	0.16	0.54
C18:1n-9	40.94	39.27
Other C18:1	1.73	1.69
C20:1	0.44	0.42
C18:2n-6	17.38	16.77
C18:3n-3	2.22	1.83
C20:5n-3	-	0.75
C22:5n-3	-	0.08
C22:6n-3	-	0.55

Results of duplicate analysis are expressed as g/100 g total fatty acids.

### Fatty acid composition of the gastric content of rat pups at day 14

The analysis of the fatty acid composition of gastric content of rat pups is commonly used to assess the fatty acid composition of the maternal milk [10]. The detailed fatty acid distribution in the different groups corresponding to neonates from mother fed with the ALA or n-3 LC-PUFA diets is provided in Table 4. Statistical analysis revealed that rat pups from ALA group received more medium chain fatty acids C8:0 (+23%, P = 0.002) and C10:0 (+40%, P = 0.001), palmitic (C16:0) acid (+11%, P = 0.001), palmitoleic (C16:1 n-9) acid (+46%, P = 0.006), C22:4 n-6 (n-6 DTA, +125%, P = 0.001) and C22:5 n-6 (n-6 DPA, +200%, P = 0.003) from maternal milk compared to n-3 LC-PUFA group (Table 4). Conversely and as expected, the gastric content of the pups from the n-3 LC-PUFA group received more EPA (+83%, P = 0.0003), C22:5 n-3 (n-3 DPA, +68%, P < 0.0001) and DHA (+65%, P < 0.0001) than pups from the ALA group. In addition, the levels of stearic (C18:0) acid (+16%, P = 0.002) and ALA (+24%, P = 0.007) were significantly higher in the n-3 LC-PUFA group.

**Table 4:** Fatty acid composition of the gastric content of rat pups at 14 days of age (g/100 g total fatty acids).

	ALA	n-3 LC-PUFA	
	Mean ± SD		P values
C6:0	0.20 ± 0.03	0.22 ± 0.02	n.s.
C8:0	4.17 ± 0.25	3.38 ± 0.17	0.002
C10:0	10.44 ± 0.79	7.43 ± 0.65	0.001
C12:0	9.41 ± 0.59	9.93 ± 0.32	n.s.
C14:0	6.75 ± 0.50	6.40 ± 0.41	n.s.
C15:0	0.11 ± 0.01	0.12 ± 0.02	n.s.
C16:0	12.93 ± 0.14	11.65 ± 0.43	0.001
C17:0	0.12 ± 0.01	0.14 ± 0.01	0.031
C18:0	5.01 ± 0.19	5.99 ± 0.35	0.002
C20:0	0.14 ± 0.04	0.19 ± 0.02	n.s.
C22:0	0.07 ± 0.02	0.03 ± 0.01	0.017
C24:0	0.04 ± 0.01	0.06 ± 0.02	n.s.
C14:1	0.02 ± 0.00	0.02 ± 0.00	n.s.
C16:1	0.98 ± 0.13	0.67 ± 0.08	0.006
C17:1	0.06 ± 0.00	0.08 ± 0.01	0.050
C18:1n-9	30.40 ± 1.38	32.97 ± 0.41	0.012
Other C18:1	1.82 ± 0.44	2.82 ± 0.11	0.005
C20:1	0.58 ± 0.12	0.44 ± 0.04	n.s.
C22:1	0.04 ± 0.01	0.04 ± 0.00	n.s.
C24:1	0.04 ± 0.04	0.03 ± 0.00	n.s.
C18:2n-6	13.65 ± 0.53	13.29 ± 0.21	n.s.
C20:2n-6	0.33 ± 0.04	0.28 ± 0.02	n.s.
C20:4n-6	0.80 ± 0.15	0.51 ± 0.07	0.011
C22:2n-6	0.04 ± 0.01	0.06 ± 0.01	0.013
C22:4n-6	0.18 ± 0.02	0.08 ± 0.02	0.001
C22:5n-6	0.09 ± 0.02	0.03 ± 0.01	0.003
C18:3n-3	0.99 ± 0.13	1.31 ± 0.09	0.008
C20:3n-3	0.04 ± 0.01	0.04 ± 0.01	n.s.
C20:5n-3	0.09 ± 0.01	0.52 ± 0.11	0.0003
C22:5n-3	0.11 ± 0.02	0.34 ± 0.03	< 0.0001
C22:6n-3	0.33 ± 0.04	0.93 ± 0.08	< 0.0001

### Fatty acid profile of brain glial cells PE and PS at day 14: bivariate statistics

The fatty acid composition of the PE classes (left columns in Table 5) was characterized by a high level of ARA and DHA that represented together more than 40% of total fatty acids. The level of ARA, and n-6 DPA found in rat neonates from dams fed with n-3 LC-PUFA was lower compared to the ALA group (P < 0.001). The most important variation observed was the increase of DHA (+18%, P = 0.0001) and dihomo-γ-linolenic acid (C20:3 n-6, DGLA) (+22%, P = 0.0018) in the n-3 LC-PUFA group. The level of dimethylacetals (DMA) that derived from the *Sn*-1 vinyl-ether residues in PE plasmalogens was higher in rat neonate from the n-3 LC-PUFA group compared to the other group of animals (+12%, P = 0.0155).

**Table 5:** Effect of maternal dietary -linolenic acid (ALA) compared to n-3 LC-PUFA (EPA+DHA) on the polyunsaturated fatty acid and DMA composition of phosphatidylethanolamine (PE) and phosphatidylserine (PS) in brain glial cells of pups (g/100 g total fatty acids).

	Phosphatidylethanolamine (PE)			Phosphaptidylserine (PS)		
	**Experimental group**			**Experimental group**		
	**ALA**	**n-3 LC-PUFA**		**ALA**	**n-3 LC-PUFA**	

	**Mean ± SD**		**P values**	**Mean ± SD**		**P values**

C18:2n-6	1.04 ± 0.12	1.17 ± 0.07	n.s.	0.46 ± 0.05	0.55 ± 0.18	n.s.
C20:2n-6	0.35 ± 0.03	0.32 ± 0.10	n.s.	0.32 ± 0.03	0.32 ± 0.08	n.s.
C20:3n-6	0.73 ± 0.01	0.93 ± 0.02	0.0018	0.77 ± 0.11	0.77 ± 0.09	n.s.
C20:4n-6	20.16 ± 0.84	17.34 ± 0.45	< 0.0001	7.93 ± 0.32	7.38 ± 0.19	n.s.
C22:4n-6	7.17 ± 0.49	6.99 ± 0.27	n.s.	6.53 ± 0.30	5.94 ± 0.71	n.s.
C22:5n-6	2.71 ± 0.21	1.64 ± 0.07	0.0003	4.07 ± 0.48	2.14 ± 0.29	< 0.0001
C18:3n-3	0.11 ± 0.07	0.12 ± 0.05	n.s.	0.16 ± 0.02	0.19 ± 0.03	n.s.
C20:5n-3	0.12 ± 0.07	0.11 ± 0.05	n.s.	0.18 ± 0.03	0.11 ± 0.05	n.s.
C22:5n-3	0.65 ± 0.02	0.80 ± 0.06	n.s.	1.07 ± 0.10	0.93 ± 0.16	n.s.
C22:6n-3	22.89 ± 0.60	27.83 ± 0.72	0.0001	26.15 ± 0.73	30.12 ± 2.27	0.0009
DMA	8.29 ± 0.34	9.40 ± 0.79	0.0155	2.18 ± 0.49	1.57 ± 0.52	n.s.

Data obtained from four pools of 6 brains excised from rat pups at 2 weeks of age.

The fatty acid composition of the PS classes (right columns in Table 5) was characterized by high levels of ARA, n-6 DTA and DHA. The level of DHA was significantly increased in the n-3 LC-PUFA group (+15%, P = 0.0009) while the level of n-6 DPA was much lower in the LC-PUFA group (-47%, P < 0.0001) compared to the ALA group. The levels of other n-6 LC-PUFA were not significantly different between groups but the n-3 to n-6 ratio was significantly higher (1.84 ± 0.11) in rat neonates from dams fed with n-3 LC-PUFA compared to the ALA group (1.37 ± 0.06, P = 0.0004, data not shown).

### Fatty acid profile of brain glial cell PE and PS at day 14: PCA analyses

**Figure 1:**
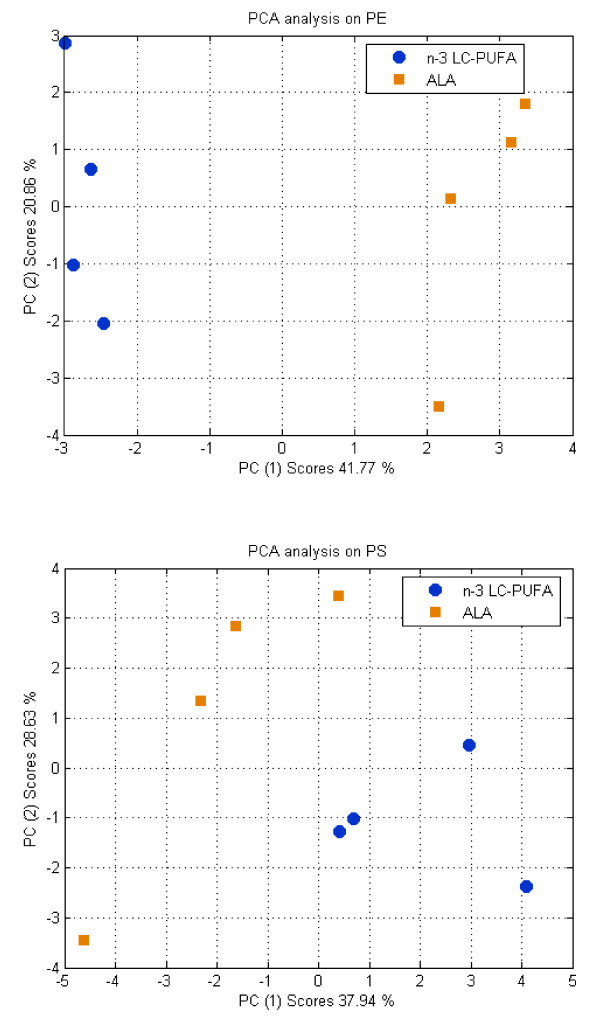
**PCA scores plot**. Discrimination between n-3 LC-PUFA and ALA groups based on phosphatidylethanolamine (PE) and phosphatidylserine (PS) fatty acid profiles.

The fatty acid composition of the PE was analyzed by PCA. Data were arranged in a set of rows (samples) and columns (variables). Variables contained quantitative values of the different fatty acids (data expressed as g per 100 g of fatty acid) including DMA (sum of individual DMA). Afterwards, data were arranged in a second time, for both n-3 LC-PUFA and ALA group samples, to help in the determination of the differences between the two groups. The data set obtained was analyzed by means of PCA. Data were pre-processed, each variable was centred and scaled to unit standard deviation in order to harmonize the contribution of all the variables. The optimal number of parameters of the model (Principal Components) was obtained after internal cross-validation. In these conditions, a one principal component model was determined to be optimal. This component explained a 41.77% of total variance in the data (Figure 1A). The second principal component explained intra-class variation and accounted for 20.86% of total variance (Figure 1A).

The analysis of the PCA scores of the first principal component 1 (PC1) showed a clear discrimination between both groups of samples issued from n-3 LC-PUFA and ALA groups (Figure 1A). To determine the contribution of the variables to this model, PC1 loadings were analyzed (Table 6). This analysis highlighted the major contribution of C14:0, C20:0, C24:0, C18:2, C20:3 n-6, C22:5 n-3, C22:6 n-3, and DMA to the profile representative of n-3 LC-PUFA class. On the contrary, compounds like C18:0, C18:1, C20:4 n-6, and C22:5 n-6 were relevant for the characterization of the ALA group.

**Table 6:** PCA Loading values for models on PE (41.77% total X-Variance) and PS (37.94% total X-Variance, PC1/28.63% total X-Variance, PC2) data.

Fatty acids	PE Data		PS Data		
	X-Loading (PC1)	Group	X-Loading (PC1)	X-Loading (PC2)	Group
C14:0	0.18	n-3 LC-PUFA	0.28	0.10	ALA
C16:0	-0.16	ALA	0.16	0.09	ALA
C17:0	0.10	n-3 LC-PUFA	0.23	0.25	
C18:0	-0.32	ALA	-0.14	0.26	n-3 LC-PUFA
C20:0	0.28	n-3 LC-PUFA	0.26	0.02	ALA
C22:0	0.06	n-3 LC-PUFA	0.19	-0.22	
C24:0	0.24	n-3 LC-PUFA	0.29	0.10	
C16:1	0.06	n-3 LC-PUFA	0.04	0.34	n-3 LC-PUFA
C18:1	-0.24	ALA	0.18	0.23	
C20:1	-0.04	ALA	0.32	-0.15	ALA
C18:2 n-6	0.21	n-3 LC-PUFA	0.00	0.31	n-3 LC-PUFA
C20:2 n-6	-0.03	ALA	0.17	0.33	
C20:3 n-6	0.33	n-3 LC-PUFA	0.07	0.11	
C20:4 n-6	-0.32	ALA	0.17	-0.26	ALA
C22:4 n-6	-0.07	ALA	0.04	-0.36	
C22:5n-6	-0.33	ALA	0.27	-0.24	ALA
C18:3 n-3	0.02	n-3 LC-PUFA	-0.10	0.34	n-3 LC-PUFA
C20:5 n-3	-0.03	ALA	0.32	0.04	ALA
C22:5n-3	0.31	n-3 LC-PUFA	0.29	0.11	ALA
C22:6 n-3	0.33	n-3 LC-PUFA	-0.32	-0.02	n-3 LC-PUFA
DMA	0.24	n-3 LC-PUFA	0.25	-0.08	ALA

Following the same internal validation procedure as for PE data, PCA on PS data showed a model with two principal components, thus reflecting some more complex structure. As shown in Figure 1B, discrimination was also possible along the PC1 (37.94% of total variance), but now with some relevant information explained by PC2 (28.63% of total variance). In this case, the analysis of the influence of the variables on the model was done by plotting the loadings of the first two components (Table 6). Accordingly, the n-3 LC-PUFA group was mainly characterized by its contents in C18:0, C16:1, C18:2 n-6, C18:3 n-3 and C22:6 n-3. Alternatively, fatty acids such as C14:0, C16:0, C20.0, C20:1, C20:5 n-3, C22:0, C20:4 n-6, C22:5 n-6, C22:5 n-3 and DMA explained the variability observed in the ALA group.

### Fatty acid profile of brain glial cells PE and PS at day 14: Correlation analyses

**Figure 2:**
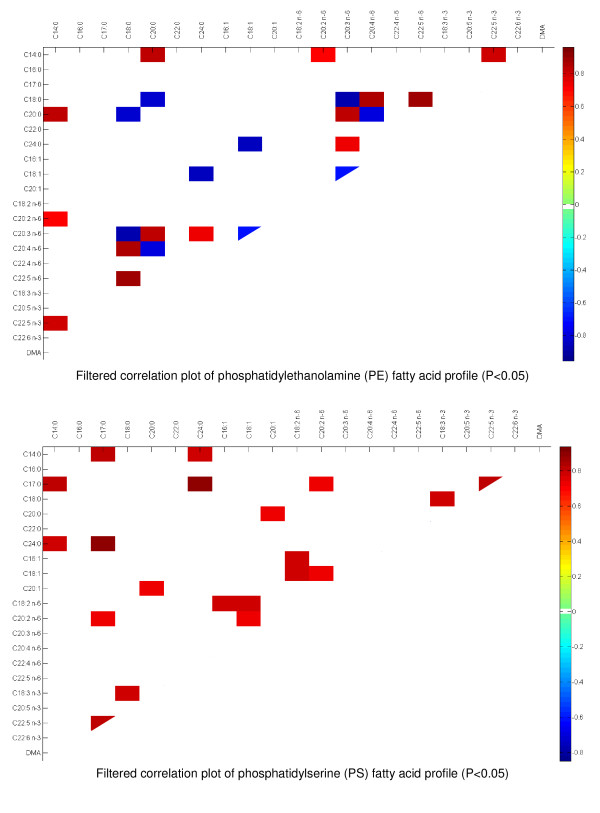
**Correlation matrix on phosphatidylethanolamine (PE) and phosphatidylserine (PS) fatty acid profiles**. Results filtered according to significance level (P < 0.05).

Correlation analysis was applied to PE and PS data to identify possible correlations between fatty acids from the brain glial cell data set. To discard non-significant associations, filtering based on significance at P < 0.05 was applied to the correlation coefficients (Figure 2A).

From the filtered correlation matrix, it appears that in the PE data set, DHA (-0.9404), n-3 DPA (-0.8385) but also DGLA (-0.9366) are negatively correlated with ARA. DGLA is positively correlated with n-3 DPA (0.8937) and DHA (0.9618). As expected, n-3 DPA and DHA are also positively correlated by each others (0.8909). ARA is negatively correlated with its precursor C18:2 n-6 (-0.7602) and positively correlated with its elongation/desaturation product n-6 DPA, (0.9394). From this analysis, it is also possible to identify positive correlation between EPA and its precursor ALA (0.8722) but also with C20:2 n-6 (0.7392). This analysis allowed to identify significant negative correlations between the level of DMA (plasmalogen derivative) and n-6 DPA (-0.783) and C18:0 (-0.718).

PS fatty acid profile data set was also processed and data were filtered based on the significance level at P < 0.05. The analysis (Figure 2B) shows less correlation and the significant ones were different when compared to the PE data set. It was possible to determine that ARA and DHA provide significant correlations (0.7218 and -0.7422, respectively) with serine derived plasmalogen DMA. In addition, EPA was found to be positively correlated with n-3 DPA and negatively correlated with DHA (0.9372, -0.8364).

## Discussion

In the present study, the effect of ALA supplementation has been not studied using a control group depleted in n-3 fatty acids. The effect of various amount of ALA on fatty acid distribution in rat neonates had been already studied and it has been clearly demonstrated that ALA supplementation does not significantly modify the level of the main n-3 LC-PUFA in brain glial cell PS and PE [10]. The main objective of the study was to compare the effect of ALA and n-3 LC-PUFA supplementation on the fatty acid composition of brain glial cells PS and PE with emphasis on n-3 LC-PUFAs such as DHA and DMAs.

It has been recently shown that DHA may act as synaptic regulator in astrocytes [24] and therefore its level might influence important function such as neurotransmission which is a key function of glial cells [24,10]. DHA accretion in PE and PS brain glial cells is crucial during the first two weeks of life in rats [10]. The metabolism of PE and PS in neural cells has been extensively investigated over the past few years and it has been shown that PE can be converted into PS (and *vice et versa*) and that DHA containing PS species are involved in neuronal survival and differentiation [see reference for an extensive review [25].

As described in the literature [10], the analysis of the gastric content of pups sacrificed at day 14 revealed that mother milk derived from dams fed with n-3 LC-PUFA contain higher level of n-3 fatty acids (ALA, EPA, n-3 DPA and DHA) than milk deriving from dams fed only ALA (Table 4). The level of n-3 fatty acids was two times higher in the gastric content of rat pups in the n-3 LC-PUFA group than in the ALA group. In the ALA group, the n-3 LC-PUFA in the gastric content derived from the maternal conversion of ALA and in some extend may originate from n-3 LC-PUFA stores in maternal adipose tissues. However, in the n-3 LC-PUFA group, DHA and EPA mainly derived from the maternal diet. Therefore, the ALA supplied through the maternal diet was not extensively metabolized which results in a higher level of ALA in the gastric content compared to the ALA group (Table 4). The transfer of n-3 fatty acids and especially DHA from the dams to the pups was higher in the n-3 LC-PUFA group and can explain the higher DHA level observed in brain glial cell PE and PS (Table 5).

The PCA analyses performed on the PE and PS fatty acid profiles clearly demonstrated how the lipid composition of maternal diet and especially the supply with different n-3 fatty acids might impact the FA composition of the brain glial cell PE and PS of rat neonates (Figure 1). The effect of supplementation with DHA on the balance between n-6 and n-3 fatty acid and especially the level of ARA and n-6 DPA in various tissues is well documented [2,10,26]. The present approach combining bivariate and multivariate statistics allowed to identify discrete correlations between the different PE and PS residues (Figure 2). We observed numerous correlations between the n-3 and n-6 LC-PUFA in the PE data set. The PE correlation matrix clearly demonstrated that n-3 LC-PUFA are negatively correlated with ARA and its elongation/desaturation product n-6 DPA but positively correlated to DGLA as previously reported in the serum and the retina [27,28]. ARA and EPA are formed from DGLA and 20:4 n-3, respectively through a Δ5 desaturase activity. It is well known EPA inhibits Δ5 enzyme activity which is necessary to convert DGLA into ARA [27,28]. It might explain why n-6 PUFA are favored whereas those formed downstream are limited when EPA is provided by the diet. This analysis also confirmed that n-6 DPA and DHA are negatively correlated (-0.9476); which confirms existing literature showing that n-6 DPA is a good marker of DHA deficiency [10,25,28,29]. Interestingly, the correlation analysis revealed that the level of plasmalogen residues (plasmenylethanolamine DMA) was negatively correlated with n-6 LC-PUFA (Figure 2), but not significantly correlated with any n-3 LC-PUFA. DMA represent about 8-9% of total PE residues (Table 5). It has been shown that deficiency of n-3 fatty acids in the diet may result in plasmalogen deficiency associated with abnormal signal transduction process in neural membranes [30]. Conversely, dietary supplementation with DHA to Zellweger patients whose tissues are strongly deficient in DHA [31] increased plasmalogen levels in erythrocytes [32]. The present observation might indicate that n-6 DPA level can potentially be used as an indicator of plasmenylethanolamine (PE plasmalogen) deficiency in brain glial cells.

The results of the analysis of the PS correlation matrix showed no correlation between n-6 and n-3 LC-PUFA (see the blank squared areas in the correlation matrix). EPA was positively correlated with its metabolite n-3 DPA but not with the end product DHA. Fatty acids up-taken from the general circulation or *de novo *synthesized were incorporated in PE but as well in phosphatidylcholine (PC) by deacylation/reacylation reaction [25]. However, PS are selectively produced from PE by serine exchange-catalyzed reaction [25].

The only noticeable correlation observed in the PS dataset is related to DMA level that are negatively correlated with DHA (-0.7422) and positively with ARA (0.7218). This might indicate that serine plasmalogen might contain significant levels of ARA in the *Sn*-2 position of the phosphatidylglycerol backbone. DMA represent 1.5 to 2.2% of the residue found to be linked to PS in brain glial cells (Table 5). Few reports mention the occurrence of serine plasmalogen in human erythrocytes [33,34] but, to our knowledge, serine plasmalogens have never been characterized in neural cells. It appears from these analyses that ethanolamine and serine plasmalogen might differ in their compositions and subsequently, one can hypothesize that they might differ in their metabolism and function.

The different phases of brain development occur differently in rats and humans. At birth the first two steps of early gliogenesis and macroneurogenesis are complete in rats whereas they happen during the first six months of gestation in humans [1]. It has been demonstrated in newborn rats that the DHA requirements at 2 weeks of age are very important [10]. This period corresponds to the microneurogenesis, late gliogenesis and microneurogenesis. At this stage, the level of DHA accretion is high in whole brain glial cell phospholipids as well as in isolated PE and PS [10]. It has been clearly shown that dietary DHA is more efficient than dietary ALA to sustain this demand [10]. Our results confirm that early supplementation of maternal diet with n-3 LC-PUFA affects the fatty acid composition of PE and PS brain glial cells during their initial developmental steps.

## Conclusion

The results of the present study confirm that the lipid composition of the maternal diet during gestation and lactation have a significant impact on the fatty acid composition of brain glial cell PE and PS in the neonates. The combination of multivariate and bivariate statistics allowed to better understand how n-3 fatty acids supplied in the maternal diet as precursor (ALA) or LC-PUFA (EPA and DHA) influence the fatty acid profile of brain glial cell PE and PS in the neonates. In PE, n-3 and n-6 LC-PUFA are correlated, consistently with literature but the correlation analysis of PS residue profile did not show any correlation showing their distinct metabolic pathways. Negative correlation between n-6 DPA, a conventional marker of DHA deficiency, and DMA in PE showed that potentially n-6 DPA can be considered as a potent marker of ethanolamine plasmalogen. In addition, we observed in this study that serine plasmalogen occur in brain glial cells of rat neonates and that the level of DMA residue is negatively correlated with DHA but positively correlated with ARA. This might indicate that serine plasmalogen might contain significant level of ARA in the *Sn*-2 position of the phosphatidylglycerol backbone.

## List of abbreviations 

PL: phospholipid; PE: phosphatidylethanolamine; PS: phosphatidylserine; DHA: docosahexaenoic acid; ALA: alpha-linolenic acid; EPA: eicosapentaenoic acid: LC-PUFA: long-chain polyunsaturated fatty acid; ARA: arachidonic acid; n-6 DPA: n-6 docosapentaenoic acid; n-3 DPA: n-3 docosapentaenoic acid; n-6 DTA: n-6 docosatetraenoic acid; DMA: dimethylacetal; DGLA: dihomo(γ)linolenic acid; PCA: principle component analysis.

## Competing interests

The authors declare that they have no competing interests.

## Authors' contributions

The present research was supported by the Nestlé Research Center. LB, FDe, NA, CJ, FJ, J-BB., CH and FDi designed and directed the study. LB, NA, CJ, FJ and BP conducted the animal trial and the analysis. LB, IM and SR performed the statistical analysis and all the authors were implicated in drafting and finalizing the paper.

## References

[B1] Morgane PJ Austin-Lafrance R Bronzino J Tonkiss J Diaz-Cintra S Cintra L Kemper T Galler JR Prenatal malnutrition and development of the brain Neuroscience and Biobehavioral Reviews 1993 17 91 128 10.1016/S0149-7634(05)80234-9 8455820

[B2] Innis S Perinatal biochemistry and physiology of long-chain polyunsaturated fatty acids J Pediatr 2003 143 S1 S8 14597908 10.1067/s0022-3476(03)00396-2

[B3] Neuringer M Jeffrey BG Visual development: neural basis and new assessment methods J Pediatr 2003 143 S87 S95 10.1067/S0022-3476(03)00406-2 14597918

[B4] Hoffman DR Birch EE Castaneda YS Fawcett SL Wheaton DH Birch DG Uauy R Visual function in breast-fed term infants weaned to formula with or without long-chain polyunsaturates at 4 to 6 months: a randomized clinical trial J Pediatr 2003 142 669 677 10.1067/mpd.2003.213 12838196

[B5] Serhan C Savill J Resolution of inflammation: the beginning programs the end Nature Imm 2005 6 1191 1197 10.1038/ni1276 16369558

[B6] Bazan NG Cell survival matters: docosahexaenoic acid signaling, neuroprotection and photoreceptors Trends Neurosci 2006 29 263 271 10.1016/j.tins.2006.03.005 16580739

[B7] Bazan NG The onset of brain injury and neurodegeneration triggers the synthesis of docosanoid neuroprotective signaling Cell Mol Neurobiol 2006 26 901 913 10.1007/s10571-006-9064-6 16897369 PMC11520625

[B8] Goyens PL Spilker ME Zock PL Katan MB Mensink RP Conversion of alpha-linolenic acid in humans is influenced by the absolute amounts of alpha-linolenic acid and linoleic acid in the diet and not by their ratio Am J Clin Nutr 2006 84 44 53 16825680 10.1093/ajcn/84.1.44

[B9] Su HM Huang MC Saad NM Nathanielsz PW Brenna JT Fetal baboons convert 18:3n-3 to 22:6n-3 in vivo. A stable isotope tracer study J Lipid Res 2001 42 581 586 11290830

[B10] Bowen RA Clandinin MT Maternal dietary 22:6n-3 is more effective than 18:3n-3 in increasing the 22:6n-3 content in phospholipids of glial cells from neonatal rat brain Br J Nutr 2005 93 601 611 10.1079/BJN20041390 15975158

[B11] de Groot RH Hornstra G van Houwelingen AC Roumen F Effect of alpha-linolenic acid supplementation during pregnancy on maternal and neonatal polyunsaturated fatty acid status and pregnancy outcome Am J Clin Nutr 2004 79 251 260 14749231 10.1093/ajcn/79.2.251

[B12] Al MD van Houwelingen AC Hornstra G Long-chain polyunsaturated fatty acids, pregnancy, and pregnancy outcome Am J Clin Nutr 2000 71 285S 91S 10617984 10.1093/ajcn/71.1.285s

[B13] McCann JC Ames BN Is docosahexaenoic acid, an n-3 long-chain polyunsaturated fatty acid, required for development of normal brain function? An overview of evidence from cognitive and behavioral tests in humans and animals Am J Clin Nutr 2005 82 281 295 16087970 10.1093/ajcn.82.2.281

[B14] Koletzko B Lien E Agostoni C Böhles H Campoy C Cetin I Decsi T Dudenhausen JW Dupont C Forsyth S Hoesli I Holzgreve W Lapillonne A Putet G Secher NJ Symonds M Szajewska H Willatts P Uauy R The roles of long-chain polyunsaturated fatty acids in pregnancy, lactation and infancy: review of current knowledge and consensus recommendations J Perinat Med 2008 36 5 14 10.1515/JPM.2008.001 18184094

[B15] Judge MP Harel O Lammi-Keefe CJ Maternal consumption of a docosahexaenoic acid-containing functional food during pregnancy: benefit for infant performance on problem-solving but not on recognition memory tasks at age 9 mo Am J Clin Nutr 2007 85 1572 1577 17556695 10.1093/ajcn/85.6.1572

[B16] Moore SA Polyunsaturated fatty acid synthesis and release by brain-derived cells in vitro J Mol Neurosci 2001 16 195 200 10.1385/JMN:16:2-3:195 11478374

[B17] Sellinger OZ Azcurra JM Marks N, Rodnight R Bulk separation of neuronal cell bodies and glial cells in the absence of added digestive enzymes Research Methods in Neurochemistry New York. Plenum Press pp3 38

[B18] Folch J Lee M Sloane-Stanley GH A simple method for the isolation and purification of total lipides from animal tissues J Biol Chem 1957 226 497 506 13428781

[B19] Leseigneur-Meynier A Gandemer G Marion D Fractionnement en classes de lipides alimentaires par HPLC à l'aide d'un détecteur à diffusion de lumière Actes du Congrès International Chevreul pour l'Etude des Corps Gras 1989 Ed ETIG, Paris 1-311-318

[B20] Glass RL Alcoholysis, Saponification and the Preparation of Fatty Acid Methyl Esters Lipids 1971 6 919 925 10.1007/BF02531175

[B21] Morrisson WR Smith LM Preparation of fatty acid methyl ester and dimethyl-acetals from lipids with boron fluoride-methanol J Lipid Res 1964 5 600 608 14221106

[B22] Bretillon L Destaillats F Joffre F Joffre C Acar N Bezelgues JB Schnebelen C Bron AM Creuzot-Garcher CP Pasquis B Maternal n-3 long chain polyunsaturated fatty acids increase n-3 fatty acids in brain glial cells and retina in the rat and improve rod sensitivity Invest Ophthalmol Vis Sci 2007 48 E-Abstract 2929

[B23] Fournier V Destaillats F Hug B Golay PA Joffre F Juanéda P Sémon E Dionisi F Lambelet P Sébédio JL Berdeaux O Quantification of eicosapentaenoic and docosahexaenoic acid geometrical isomers formed during fish oil deodorization by gas-liquid chromatography J Chromatogr A 2007 1154 353 359 10.1016/j.chroma.2007.03.099 17449039

[B24] Grintal B Champeil-Potokar G Lavialle M Vancassel S Breton S Denis I Inhibition of astroglial glutamate transport by polyunsaturated fatty acids: evidence for a signalling role of docosahexaenoic acid Neurochem Int 2009 54 535 543 10.1016/j.neuint.2009.02.018 19428799

[B25] Kim HY Novel metabolism of docosahexaenoic acid in neural cells J Biol Chem 2007 282 18661 18665 10.1074/jbc.R700015200 17488715

[B26] Galli C Trzeciak HI Paoletti R Effects of dietary fatty acids on the fatty acid composition of brain ethanolamine phosphoglyceride: reciprocal replacement of n6 and n3 polyunsaturated fatty acids Biochim Biophys Acta 1971 248 449 454

[B27] Barham JB Edens MB Fonteh AN Johnson MM Easter L Chilton FH Addition of eicosapentaenoic acid to γ-linolenic acid supplemented diets prevents serum arachidonic acid accumulation in humans J Nutr 2000 130 1925 1931 10917903 10.1093/jn/130.8.1925

[B28] Schnebelen C Viau S Gregoire S Joffre C Creuzot-Garcher CP Bron AM Bretillon L Acar N Nutrition for the eye: different susceptibility of the retina and the lacrimal gland to dietary omega-6 and omega-3 polyunsaturated fatty acid incorporation Ophthalmic Res 2009 41 216 224 10.1159/000217726 19451735

[B29] Levant B Ozias MK Jones KA Carlson SE Differential effects of modulation of docosahexaenoic acid content during development in specific regions of rat brain Lipids 2006 41 407 14 10.1007/s11745-006-5114-6 16933785

[B30] Farooqui AA Horrocks LA Plasmalogens: workhorse lipids of membranes in normal and injured neurons and glia Neuroscientist 2001 7 232 45 10.1177/107385840100700308 11499402

[B31] Martinez M Abnormal profiles of polyunsaturated fatty acids in the brain, liver, kidney and retina of patients with peroxisomal disorders Brain Res 1992 583 171 182 1504825 10.1016/s0006-8993(10)80021-6

[B32] Martinez M Restoring the DHA levels in the brains of Zellweger patients J Mol Neurosci 2001 16 309 316 10.1385/JMN:16:2-3:309 11478386

[B33] Dodge JT Phillips GB Composition of phospholipids and of phospholipid fatty acids and aldehydes in human red cells J Lipid Res 1967 8 667 671 6057495

[B34] Farquhar JW Human erythrocyte phosphoglycerides I. Quantification of plasmalogens, fatty acids and fatty aldehydes Biochim Biophys Acta 1962 60 80 89 10.1016/0006-3002(62)90374-8 13891677

